# Enlarged glycemic variability in sulfonylurea-treated well-controlled type 2 diabetics identified using continuous glucose monitoring

**DOI:** 10.1038/s41598-021-83999-z

**Published:** 2021-03-01

**Authors:** Fumi Uemura, Yosuke Okada, Keiichi Torimoto, Yoshiya Tanaka

**Affiliations:** First Department of Internal Medicine, School of Medicine, University of Occupational and Environmental Health, Japan, 1-1 Iseigaoka, Yahatanishi-ku, Kitakyushu, 807-8555 Japan

**Keywords:** Diabetes, Type 2 diabetes

## Abstract

Time in range (TIR) is an index of glycemic control obtained from continuous glucose monitoring (CGM). The aim was to compare the glycemic variability of treatment with sulfonylureas (SUs) in type 2 diabetes mellitus (T2DM) with well-controlled glucose level (TIR > 70%). The study subjects were 123 patients selected T2DM who underwent CGM more than 24 h on admission without changing treatment. The primary endpoint was the difference in glycemic variability, while the secondary endpoint was the difference in time below range < 54 mg/dL; TBR < 54, between the SU (n = 63) and non-SU (n = 60) groups. The standard deviation, percentage coefficient of variation (%CV), and maximum glucose level were higher in the SU group than in the non-SU group, and TBR < 54 was longer in the high-dose SU patients. SU treatment was identified as a significant factor that affected %CV (β: 2.678, p = 0.034). High-dose SU use contributed to prolonged TBR < 54 (β: 0.487, p = 0.028). Our study identified enlarged glycemic variability in sulfonylurea-treated well-controlled T2DM patients and high-dose SU use was associated with TBR < 54. The results highlight the need for careful adjustment of the SU dose, irrespective of glycated hemoglobin level or TIR value.

## Introduction

Sulfonylureas (SUs) are one of the oldest oral glucose-lowering agents that have been used since the 1950s. They are useful regardless of age and body weight and have been demonstrated to prevent microangiopathies^1^. Although they are used in many patients for these reasons, SUs are associated with highest risk of hypoglycemia among the oral glucose-lowering agents. A previous study using continuous glucose monitoring (CGM) and 24-h continuous electrocardiography reported that SU-related hypoglycemia was associated with repolarization of cardiomyocytes and QTc prolongation^2^. Furthermore, cardiac diseases other than ischemic heart diseases, such as arrhythmia and heart failure, are responsible for 8.7% of deaths among patients with diabetes mellitus in Japan. These diseases are the next most common causes of death after malignant neoplasms, infections, and angiopathy^3^. Therefore, hypoglycemia should be prevented to extend healthy life expectancy during treatment of diabetes mellitus. In this regard, the CGM should be useful for monitoring glucose dynamics associated with the use of SUs.

CGM can detect hypoglycemia, especially unnoticed hypoglycemic events that occur between midnight and early morning^4,5^. We reported previously that patients with type 2 diabetes mellitus (T2DM) with low average glucose (AG) levels and large glycemic variability, as detected by CGM, are at a high risk of hypoglycemia^6^, and that large variability in blood glucose level is associated with the risk of hypoglycemia regardless of glycated hemoglobin (HbA1c) levels^7^. However, few studies have used CGM to assess glycemic variability and the risk of hypoglycemia based on the SUs-treated well-controlled T2DM.

At the 79th Scientific Sessions of the American Diabetes Association held June 7–11, 2019, the international consensus guidelines for glycemic control with CGM were issued for the first time^8^. The time in range (TIR) is defined as the percent period of time with glucose level measured by the CGM are within 70–180 mg/dL. In patients with T2DM, a TIR of > 70% was set as the management target for prevention of microangiopathies, similar to the cutoff values used for HbA1c. A subsequent US multicenter validation study using data from the Diabetes Control and Complications Trial reported that low TIR was associated with progression of retinopathy and development of microalbuminuria while higher TIR levels were associated with lesser chance of microangiopathy^9^. Thus, maintaining a favorable TIR is important for the prevention of microangiopathies. However, there are no reports on glycemic variability or the risk of hypoglycemia in patients treated with SUs who achieve TIR > 70%. This study included hospitalized T2DM patients with TIR > 70%. We investigated the characteristics of glycemic variability and compared the risk of hypoglycemia in patients treated with or without SUs.

## Methods

### Patients

**Figure 1:**
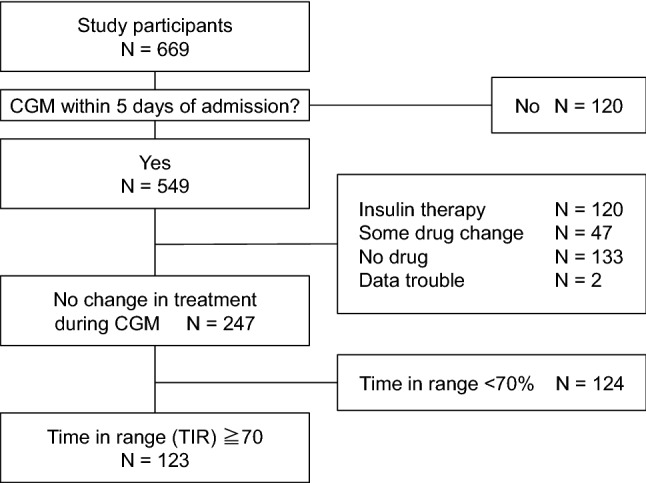
Flow diagram of the patient recruitment process.

This study was designed as a cross-sectional study. We enrolled 669 patients with T2DM who were admitted to the Hospital of the University of Occupational and Environmental Health or its affiliated hospitals for glycemic control and education on diabetes management. All patients underwent CGM between April 2010 and March 2019 (excluding those receiving steroids). We analyzed the CGM data of 247 patients except those treated with insulin and those treated with no drug who started CGM within 5 days of hospitalization and did not change the doses or preparations after wearing a CGM device. Of these 247 patients, the data of 123 patients with TIR > 70% were analyzed (Fig. 1).

The meal during CGM was adjusted to 25–30 kcal/kg of ideal body weight (60% carbohydrates, 15–20% fats, and 20–25% protein) for patients with nephropathy at stage II or earlier, and 30–35 kcal/kg of ideal body weight (60–70% carbohydrates, 15–20% fats, and 15–20% protein) for patients with nephropathy at stage III or later. The meal contents were not changed during the CGM, and the patients consumed all inpatient meals. Moreover, the amount of exercise was kept constant.

Diabetic neuropathy was defined as neuropathy meeting two of the following three criteria: (1) the presence of subjective symptoms presumably caused by diabetic polyneuropathy, (2) weak or absent Achilles tendon reflex bilaterally, and (3) decreased vibration perception at the medial malleolus bilaterally. Diabetic retinopathy was defined as the presence of any of the following fundoscopic findings: simple retinopathy, preproliferative retinopathy, and proliferative retinopathy. Diabetic nephropathy was defined as a urinary albumin to creatinine ratio ≥ 30 mg/g, the presence of overt proteinuria, or an estimated glomerular filtration rate (eGFR) < 30 mL/min/1.73 m^2^. Micro-angiopathy was defined as the presence of any of the following conditions: neuropathy, retinopathy, or nephropathy. Macroangiopathy was defined as the presence of history of any of the following conditions: ischemic heart disease, stroke, or arteriosclerosis obliterans.

This study was conducted in accordance with the Declaration of Helsinki and the current applicable ethical codes. The study protocol was approved by the ethics committees of the Hospital of the University of Occupational and Environmental Health and its affiliated hospitals (Trial registration: H27-186, Registered 25 December 2015). This research is registered in University Medical Information Network [UMIN] ID: UMIN000025433. Before the start of the study intervention, all patients received information on the study and provided informed consent.

### CGM system

The CGM devices used were Gold (CGMS System Gold, Medtronic Inc.; Fridley, MN) and iPro2 (Medtronic MiniMed Inc.; Northridge, CA). These CGM devices continuously measure interstitial glucose levels within the range of 40–400 mg/dL. The sensor placed in the subcutaneous tissue converts the interstitial glucose levels into electrical signals and records measurements every 5 min, up to a maximum of 288 measurements per day. Interstitial glucose levels measured by the CGM devices have been reported to correlate well with venous blood glucose levels^10^. The CGM data used in the present study were calibrated before each of the three meals and at bedtime with data from a self-monitored blood glucose device (MEDISAFE MINI; Terumo, Inc.).

We analyzed 288 measurements taken between 00:00 and 24:00 after placement of the CGM device for 24 h. The CGM parameters included average glucose (AG); standard deviation (SD); percentage coefficient of variation (%CV); maximum glucose level; minimum glucose level; large amplitude of glycemic excursions (LAGE); mean postprandial glucose excursion (MPPGE) following breakfast, lunch, and supper; low blood glucose index (LBGI); high blood glucose index (HBGI); time above range (TAR, defined as the percent time with glucose level above 180 mg/dL); TIR (defined as the percent time with glucose level between 70 and 180 mg/dL); time below range < 70 mg/dL (TBR < 70, defined as the percent of time with glucose level less than 70 mg/dL); time below range < 54 mg/dL (TBR < 54, defined as the percent time with glucose level less than 54 mg/dL). %CV was calculated by the following formula: (SD/AG) × 100. LAGE was calculated as the difference between the maximum and minimum glucose levels. To calculate MPPGE, preprandial glucose levels were measured at 07:00 for breakfast, 12:00 for lunch, and 18:00 for supper, and the corresponding postprandial levels were measured from 07:00 to 12:00, from 12:00 to 18:00, and from 18:00 to 24:00, respectively.

Then, the difference between the maximum postprandial glucose level and the preprandial glucose level for each meal was calculated as MPPGE. In addition, hypoglycemia was defined as glucose level < 70 mg/dL as measured by CGM, and severe hypoglycemia was defined as glucose level < 54 mg/dL.

### Measurements of biochemical variables

HbA1c levels (%) were measured on admission and converted to National Glycohemoglobin Standardization Program (NGSP) values for assessment. The HbA1c levels measured as Japan Diabetes Society (JDS) values were converted using the following formula: HbA1c (NGSP) (%) = HbA1c (Japan relationship of HbA1c, JDS) × 1.02 + 0.25 (%)^11^. The eGFR was calculated as follows: 194 × serum creatinine level (mg/dL) − 1.094 × age − 0.287 for men, and 194 × serum creatinine level (mg/dL)  − 1.094 × age − 0.287 × 0.739 for women.

### Statistical analysis

Data are expressed as mean ± SD. The Shapiro–Wilk test was used to test for normal distribution of data. For comparisons between two groups, we used the Student's t-test for parameters with normal distribution, and the Mann–Whitney U-test for parameters with skewed data distribution. For nominal scale, the Fisher's exact test was performed when some cells had an expected value within 5, whereas the χ^2^ test was performed otherwise. Univariate and multivariate linear regression analyses were performed to estimate the regression coefficients for %CV and TBR < 54. Since multicollinearity was observed between age and the duration of diabetes; between HbA1c and fasting blood glucose (FBG) levels; and between neuropathy, retinopathy, and nephropathy; we excluded diabetes duration, FBG, neuropathy, retinopathy, and nephropathy from the model of multivariate analysis, and included age, HbA1c and microangiopathy. In the model, age, sex, body mass index (BMI), microangiopathy, macroangiopathy, HbA1c level, eGFR, SU use, thiazolidinedione (TZD) use, biguanide (BG) use, alpha-glucosidase inhibitor (α-GI) use, glinide use, dipeptidyl peptidase-4 inhibitor (DPP4i) use, glucagon-like peptide 1 receptor agonist (GLP1RA) use, and sodium-glucose cotransporter 2 inhibitor(SGLT2i) use were entered as independent variables. In addition, we used the Fisher’s exact test to assess the association between TBR < 70 and TBR < 54 based on the use of SUs (after dividing the patients into the high-dose, recommended-dose, and non-SU users). The Statistical Program for Social Sciences version 25.0 (IBM-SPSS Statistics) was used for all statistical analyses. We determined the level of significance p < 0.05.

### Endpoints

The primary endpoint was the difference in glycemic variability, based on CGM parameters (%CV), between the SU use and non-SU use groups in patients with TIR > 70%. The secondary endpoint was the comparison of proportions of patients with severe hypoglycemia (as defined above).

## Results

### Clinical characteristics of study participants

The study included 123 patients (Fig. 1). Table 1 shows the background of study participants. The mean age was 59.9 years. BMI was 26.9 kg/m^2^. The mean duration of diabetes was 8.4 years (range, 0–44 years). The mean FBG level in the early morning on admission was 133 mg/dL, and the mean HbA1c level was 8.3%. There were no significant differences in FBG and HbA1c levels between the SU and non-SU groups. The duration of diabetes was significantly longer in the SU group than in the non-SU group; however, there was no difference with respect to the presence of diabetic complications. The number of drugs used was higher in the SU group than in the non-SU group (2.5 vs. 1.5, p < 0.001). Three types of SUs were used: glimepiride, gliclazide, and glibenclamide. Glimepiride was used by 36 patients, while glibenclamide was used by only two patients. The Japan Diabetes Society recommends reducing the dose of SUs when used in combination with incretin-related agents and SGLT2 inhibitors^12^. We defined the doses exceeding 2 mg, 40 mg, and 1.25 mg for glimepiride, gliclazide, and glibenclamide, respectively, as high dose with reference to this recommendation. Ten participants were high-dose SU users. Thirty-five patients used SUs concomitantly with incretin-related agents (without SGLT2i), of whom 33 used SUs in line with the recommended doses. There was no difference in patient background by year of admission.

**Table 1:** Clinical characteristics of study participants.

	All (n = 123)	SU group (n = 63)	Non-SU group (n = 60)	p-value
Age, years	59.9 ± 14.3	61.2 ± 12.8	58.5 ± 15.7	0.472
Gender, male/female	70/53	31/32	39/21	0.077
BMI, kg/m^2^	26.9 ± 5.8	26.9 ± 5.5	27.0 ± 6.2	0.832
Diabetes duration, year	8.4 ± 1.6	10.7 ± 8.4	7.8 ± 9.2	0.006*
**Diabetes complications**				
Neuropathy, n (%)	64 (52.0)	35 (55.6)	29 (48.3)	0.423
Retinopathy, n (%)	26 (21.1)	15 (23.8)	11 (18.3)	0.457
Nephropathy, n (%)	32 (26.0)	18 (28.6)	14 (23.3)	0.508
Micro-angiopathy, n (%)	75 (61.0)	40 (63.5)	35 (58.3)	0.558
Macro-angiopathy, n (%)	20 (16.3)	8 (12.7)	12 (20.0)	0.273
Fast blood glucose, mg/dl	132.0 ± 30.4	133.6 ± 27.1	130.4 ± 33.7	0.284
HbA1c, %	8.4 ± 1.6	8.5 ± 1.6	8.4 ± 1.5	0.658
eGFR, ml/min/1.73m^2^	75.5 ± 23.9	75.0 ± 22.7	76.0 ± 25.4	0.588
**Number of medications, n (%)**				
1 drug	48 (39.0)	36 (60.0)	12 (19.0)	< 0.001*
2 drugs	44 (35.8)	20 (33.3)	24 (38.1)	
3 drugs	30 (24.4)	4 (6.7)	26 (41.3)	
4 drugs	1 (0.8)	0 (0)	1 (1.6)	
**Medication, n (%)**				
SU	63 (51.2)	63 (100)	0 (0)	-
Glimepiride	25 (20.3)	25 (39.7)	0 (0)	-
Gliclazide	36 (29.3)	36 (57.1)	0 (0)	-
Glibenclamide	2 (1.6)	2 (3.2)	0 (0)	-
High-dose SU	10 (8.1)	10 (15.9)	0 (0)	-
TZD	21 (17.0)	11 (17.5)	10 (16.7)	0.907
BG	49 (39.8)	26 (41.3)	23 (38.3)	0.739
α-GI	13 (10.6)	7 (11.1)	6 (10.0)	0.841
Glinide	2 (1.6)	0 (0)	2 (3.3)	0.144
DPP4 inhibitor	75 (61.0)	32 (50.8)	43 (71.7)	0.018^†^
GLP1RA	4 (3.3)	3 (4.8)	1 (1.7)	0.333
SGLT2 inhibitor	1 (0.8)	0 (0)	1 (1.7)	0.488

Data are mean ± SD or n (%). P values are for differences between the SU and non-SU groups.

*p < 0.05 by Mann–Whitney’s U test.

^†^p < 0.05 by χ^2^ test.

### CGM parameters of glycemic variability

**Figure 2:**
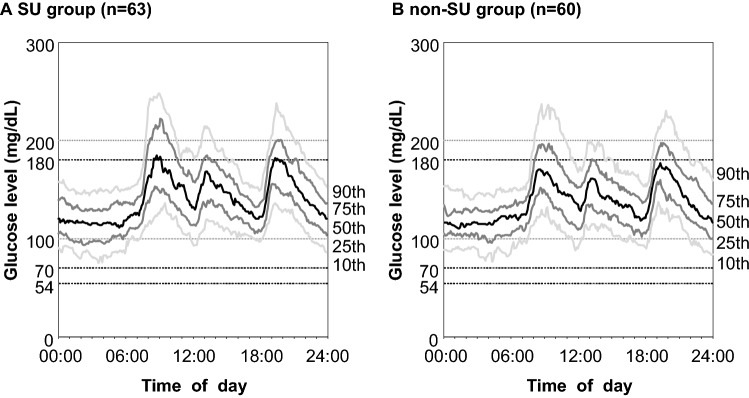
(**a**) The 24-h glycemic profile of 63 patients of the SU therapy group and (**b**) 60 patients of non-SU therapy group. Line charts are the glucose levels were measured by continuous glucose monitoring. The center solid line is the median value, while the two gray solid lines are the interquartile range (25th and 75th percentiles), and the light gray lines represent the 10th and 90th percentiles.

Table 2 and Fig. 2 show the recorded CGM parameters. The AG level for the whole group was 138.8 mg/dL, with no significant difference between the two groups. However, the SD (31.6 mg/dL vs. 27.2 mg/dL, p = 0.030), nocturnal SD (11.2 mg/dL vs. 9.4 mg/dL, p = 0.028), nocturnal %CV (10.0% vs. 7.9%, p = 0.006), maximum glucose level (223.8 mg/dL vs. 208.7 mg/dL, p = 0.020), and LAGE (132.7 mg/dL vs. 115.7 mg/dL, p = 0.020) were significantly higher in the SU group than the non-SU group. There were no significant differences in the minimum glucose levels and LBGI between the two groups. Since the present study included patients with TIR > 70%, the mean TIR for the whole group was as high as 87.2%, with no significant difference between the two groups. Although no significant differences were observed in TAR and TBR < 70 between the two groups, TBR < 54 was significantly higher in the SU group than in the non-SU group (0.22% vs. 0%, p = 0.048). The proportions of patients with TBR < 70 and TBR < 54 were not significantly different in SU users.

**Table 2:** CGMS parameters of study participants.

	All (n = 123)	SU group (n = 63)	Non-SU group (n = 60)	p-value
**AG, mg/dl**	138.8 ± 16.9	140.3 ± 15.7	137.2 ± 18.1	0.392
0:00–07:00	117.1 ± 19.8	116.9 ± 29.6	117.3 ± 19.0	0.970
07:00–24:00	147.8 ± 18.7	150.0 ± 17.1	145.5 ± 20.2	0.282
**SD, mg/dl**	29.4 ± 10.3	31.6 ± 11.0	27.2 ± 9.1	0.030*
0:00–07:00	10.3 ± 5.8	11.2 ± 5.9	9.4 ± 5.6	0.028*
07:00–24:00	28.8 ± 10.2	31.0 ± 10.8	26.5 ± 9.1	0.026*
**%CV, %**	21.2 ± 7.2	22.7 ± 7.8	19.8 ± 6.2	0.054
00:00–07:00	9.1 ± 5.6	10.0 ± 6.1	8.2 ± 5.0	0.024*
07:00–24:00	19.4 ± 6.3	20.6 ± 6.7	18.2 ± 5.6	0.051
Maximum glucose, mg/dl	216.4 ± 38.1	223.8 ± 36.2	208.7 ± 38.7	0.020*
Minimum glucose, mg/dl	92.0 ± 20.6	91.0 ± 21.7	93.0 ± 19.6	0.767
LAGE, mg/dl	124.4 ± 40.1	132.7 ± 41.2	115.7 ± 37.2	0.020*
**MPPGE, mg/dl**	65.9 ± 26.0	68.8 ± 25.4	62.8 ± 26.5	0.125
Morning	80.9 ± 35.9	86.3 ± 36.4	75.1 ± 34.7	0.069
Noon	46.3 ± 28.4	45.1 ± 29.8	47.6 ± 26.9	0.622
Evening	70.4 ± 37.0	74.8 ± 35.8	65.9 ± 38.0	0.242
LBGI	1.20 ± 2.90	1.05 ± 1.82	1.35 ± 3.72	0.736
HBGI	6.98 ± 6.21	7.46 ± 6.59	6.48 ± 5.79	0.392
Time above range, > 180 mg/dl, %	12.0 ± 9.6	13.3 ± 9.3	10.6 ± 9.9	0.111
Time in range, 70–180 mg/dL %	87.2 ± 9.8	85.7 ± 9.5	88.8 ± 9.9	0.077
Time below range, < 70 mg/dl, %	0.77 ± 2.43	0.97 ± 2.89	0.56 ± 1.83	0.487
Time below range, < 54 mg/dL, %	0.11 ± 0.61	0.22 ± 0.84	0	0.048*
Presence of TBR < 70, n (%)	17 (13.8)	10 (15.9)	7 (11.7)	0.457
Presence of TBR < 54, n (%)	4 (3.3)	4 (6.4)	0 (0)	0.119

Data are mean ± SD. P values are for the differences between the SU and non-SU groups.

*p < 0.05 by Mann–Whitney’s U test.

Univariate and multivariate linear regression analyses were performed to assess those factors associated with the primary endpoint of glycemic variability, and the regression coefficient for %CV was estimated (Table 3). Age, gender, BMI, HbA1c(NGSP), eGFR, Micro-angiopathy, Macro-angiopathy, SU, TZD, BG, α-GI, glinide, DPP4 inhibitors, GLP1RA, and SGLT2 inhibitors were fed into the model. We selected one of similar indicators from which multicollinearity may occur for each factor; neuropathy, retinopathy, and nephropathy were evaluated as micro-angiopathy. The use of SUs was identified as an important determinant. The use of SUs increased the %CV by 2.678 (95% confidence interval [CI]: 0.211–5.145). In addition, α-GI use, DPP4i use, and GLP1RA use were identified as factors that reduced %CV, whereas only SU use was identified as a factor that increased %CV.

**Table 3:** %CV analyzed by single and multiple linear regression analyses.

	Univariable linear regression			Multivariable linear regression		
	β	*SE*	P	β	*SE*	P
Age	− 0.052	0.045	0.253	− 0.063	0.054	0.244
Gender	1.23	1.306	0.348	0.948	1.26	0.453
BMI	− 0.224	0.11	0.045*	− 0.351	0.112	0.002*
HbA1c(NGSP)	0.171	0.418	0.684	− 0.327	0.423	0.442
eGFR	0.050	0.027	0.064	0.016	0.03	0.583
Micro-angiopathy	0.054	1.331	0.968	1.128	1.258	0.372
Macro-angiopathy	0.058	1.759	0.974	0.148	1.699	0.931
SU	2.886	1.272	0.025*	2.678	1.244	0.034*
TZD	1.456	1.72	0.399	0.815	1.616	0.615
BG	0.488	1.325	0.713	− 0.828	1.296	0.524
α-GI	− 8.002	1.982	< 0.001*	− 9.186	2.14	< 0.001*
Glinide	− 5.823	5.105	0.256	− 1.076	5.162	0.835
DPP4 inhibitor	− 2.021	1.318	0.128	− 3.763	1.325	0.005*
GLP1RA	− 4.649	3.635	0.203	− 8.656	3.39	0.012*
SGLT2 inhibitor	− 4.917	7.214	0.497	− 6.162	6.685	0.359
R^2^				0.320		

*β* regression coefficient, *SE* standard error.

*p < 0.05.

### CGM parameters of hypoglycemia

TBR < 54 was significantly higher in the SU than the non-SU group (0.22% vs. 0%, p = 0.048) (Table 2). Although the incidence of hypoglycemia was not significantly different, that of severe hypoglycemia was significantly higher in the SU group (4 patients vs. 0 patient). Two of these four patients used high-dose SUs. Severe hypoglycemia was observed in three patients between 00:00 and 07:00 and in one patient between after supper and midnight. In all patients who developed severe hypoglycemia during treatment with SUs, severe hypoglycemia occurred between after supper and before early morning. All patients were unaware and asymptomatic. Univariate and multivariate linear regression analyses were performed to assess the factors associated with the secondary endpoint of the duration of severe hypoglycemia, and to estimate the regression coefficient for TBR < 54, which reflects the duration of severe hypoglycemia (Supplemental Table S1). Age, gender, BMI, HbA1c(NGSP), eGFR, microangiopathy, macroangiopathy, SU, TZD, BG, α-GI, glinide, DPP4 inhibitors, GLP1RA, and SGLT2 inhibitors were fed into the model. We selected one of similar indicators from which multicollinearity may occur for each factor; neuropathy, retinopathy, and nephropathy were evaluated as micro-angiopathy. The use of SU was not identified as a determinant of TBR < 54. However, since the use of high-dose SU has been reported to cause severe hypoglycemia^13^, the same analysis was performed for the use of high-dose SU instead of SU (Supplemental Table S2). The use of high-dose SUs was identified as an important determinant. The use of high-dose SUs increased TBR < 54 by 0.487 (95% CI 0.053–0.922, p = 0.028). Analysis of data according to SU dose (high dose, recommended dose, and non-SU), TBR < 70 and TBR < 54 by Fisher’s exact test showed significant difference in TBR < 54 group (Table 4).

**Table 4:** Relationship between dose of SU and hypoglycemia.

	High dose SU group (n = 10)	Recommended dose SU group (n = 53)	non-SU group (n = 60)	P
Presence of TBR < 70, n (%)	3 (30.0)	7 (13.2)	7 (11.7)	0.265
Presence of TBR < 54, n (%)	2 (20.0)	2 (3.77)	0 (0)	0.008*

We showed the number of patients who presence of TBR < 70 and < 54. We used Fisher’s exact test. *p < 0.05.

## Discussion

This is the first report in which, despite favorable glycemic control as indicated by TIR > 70%, T2DM patients treated with SUs showed larger glycemic variability. Furthermore, high-dose SU users were found to develop more prolonged and severe hypoglycemia than non-SU users. Monnier et al.^14,15^ identified the importance of HbA1c, glycemic variability, and hypoglycemia as three factors in achieving favorable glycemic control. It considered that large glycemic variability is associated with the development of coronary artery disease^16^, and control of such variability may also contribute to the prevention of macro-angiopathies. For the optimal treatment of diabetes mellitus, it is important to take into consideration any intra- and inter-day variability in glucose level, including periods of hypoglycemia, instead of viewing glycemic control through HbA1c level alone. In the present study, we dissected the effects of SUs with a special emphasis on TIR > 70%, a newly proposed index of glycemic control. Our results showed that even in patients with TIR > 70% who met the criteria for stable glycemic control, the use of SUs seems to enhance glycemic variability and the use of high-dose SUs seems to prolong the duration of hypoglycemia. It is important to comprehensively control glucose levels based on glycemic variability and the presence or absence of hypoglycemia in consideration of the characteristics of the drugs without being limited by one criterion such as the HbA1c level or TIR.

In patients with T2DM, large swings in blood glucose levels have been reported to more likely lead to hypoglycemia irrespective of HbA1c level^6,7^. Our group also found high TBR even in insulin-treated patients with low AG level and high SD^17^. TIR is a new parameter of glycemic control, which was selected following the wide adoption of CGM in recent years and has been set as an index for the prevention of microangiopathy. However, there are no detailed reports on the risk of hypoglycemia and differences in glycemic variability in association with the use of various glucose-lowering agents in patients with favorable TIR. Among the CGM parameters, %CV is known to be associated with glycemic variability. Especially, the incidence of hypoglycemia has been reported to increase when %CV exceeds 36^18^. Our study was conducted in patients who were not treated with insulin and included only two patients with %CV > 36, both of whom used SUs. The present study demonstrated clearly that the use of SUs and high-dose SUs was associated with an increase in %CV and an incidence of severe hypoglycemia, respectively. To our knowledge, there are no detailed published studies on the use of the CGM for the assessment of intra-daily fluctuations in blood glucose level, especially in SU-treated patients with TIR > 70%. Our study is the first to report that the use of SUs seems to increase glycemic variability and high-dose SUs seems to the likelihood of prolonged severe hypoglycemia even in patients with TIR > 70%.

In the present study, the analysis of the CGM data demonstrated that SU use increased nocturnal glycemic variability and severe hypoglycemia occurred after supper until dawn. A CGM study on the use of SUs in patients with T2DM showed that the patients reached their minimum glucose level between 03:00 and 05:00 regardless of their HbA1c levels. Furthermore, it was reported that the lower their HbA1c, the longer the TBR^19^. As shown in Table 2, the SD and %CV values from 00:00 to 07:00 were significantly higher in the SU group. What is the reason for the nocturnal dip in glucose level? While the involvement of sleep apnea, hypertension, and obesity has been suggested^20^, nocturnal glycemic variability has also been reported to be associated with poor sleep quality^21^. In patients treated with SUs, large nocturnal glycemic variability may reduce the quality of sleep. Our study suggested that nocturnal glycemic variability occurs in SU-treated patients and even in those with TIR > 70%. Thus, it is important to pay attention to nocturnal glycemic variability and severe hypoglycemia in patients treated with SUs and evaluate not only TIR but also %CV, TBR < 70, and TBR < 54 when CGM is performed.

Among the three daily meals, glucose levels are most likely to increase after breakfast in patients with T2DM^22^. In addition, post-breakfast hyperglycemia occurs in patients on insulin-dependent diabetes mellitus, even in the absence of nocturnal hypoglycemia^23^. In the current study, we found TBR < 54 only in SU users; however, there was no difference between day and night. Furthermore, SU users have higher maximum glucose levels than non-SU users, suggesting an increase in postprandial glucose level due to SU. Although there was no significant difference in the increase in morning MPPGE between SU and non-SU users, Fig. 2 suggests that the post-breakfast glucose level was higher in SU users than in non-SU users. The increased nocturnal glucose variability and post-breakfast hyperglycemia cannot be explained only by the Somogyi phenomenon that may be caused by nocturnal hypoglycemia. It is supposed that the use of SU itself is related an increase in nocturnal glycemic variability and post-breakfast hyperglycemia, which could explain why LAGE, i.e., the difference between the minimum and maximum glucose levels, was higher in SU users than in non-SU users. Even in patients with well-controlled glucose levels, as demonstrated by TIR > 70%, the amplitude of increase in postprandial glucose levels after breakfast was larger in patients treated with SUs. Thus, attention should be paid to the control of postprandial hyperglycemia after breakfast.

A number of clinical studies examined the factors associated with T2DM and hypoglycemia. Hypoglycemia was reported to occur more frequently in females than males with T2DM when they were introduced to insulin^24^. Another CGM study demonstrated that males were more likely to develop hypoglycemia than females in T2DM patients on insulin therapy^25^. Thus, there is some disagreement about the relationship between hypoglycemia and gender. It has been reported that low HbA1c, old age, and a long DM disease duration are risk factors of hypoglycemia in patients on none-insulin glucose-lowering agents, and that the occurrence of mild hypoglycemia (i.e., 54–69 mg/dL) is a risk factor for severe hypoglycemia^26^. In addition, for patients with T2DM (HbA1c < 7.0%), severe hypoglycemia was dependent on excessive use of antidiabetic drugs and several other comorbidities^27^. While there is no large-scale clinical study with of CGM-monitored patients, one Japanese study reported that old age, low HbA1c, insulin injection, and use of insulin secretagogue are risks for TBR < 70^28^. In the present study, we demonstrated that the use of high-dose SU drug is associated with TBR < 54 regardless of age, gender, diabetes mellitus complication, and disease duration, even in T2DM patients with good glycemic index of “TIR > 70%”. Based on this finding, we advocate avoidance of high-dose SUs to prevent potential severe hypoglycemia, regardless of the patient background.

This study has several limitations. First, because it was a retrospective study conducted in a limited number of hospitals, the data could have been affected by unintentional selection bias. Second, because the study included only patients who underwent CGM who did not change their medications during the study, patients whose medications were changed due to the development of hypoglycemia or other reasons immediately after admission were excluded from the analyses. This was a selection bias; thus, prospective studies need to be conducted to verify the findings of the present study. Third, the patient characteristics were different between the two treatment groups. In the present study, the duration of diabetes was longer in the SU-treated patients; however, there was no difference in microvascular and macrovascular complications. Fourth, this study was conducted between 2010 and 2019 and included a small number of users of SGLT2 inhibitor, which has become available in recent years. Further studies that include more users of SGLT2 inhibitor are needed in the future due to the projected increase in use frequency. In addition, CGM data in the present study were measured only for 24 h per patient; thus, we could not assess the daily glycemic profiles. To overcome these limitations, we need to conduct a detailed study with a larger study size and longer duration of monitoring in the future.

## Conclusion

Even in T2DM patients with TIR > 70% who met the criteria for stable glycemic control, according to the newly proposed international consensus guidelines for CGM, the use of SUs increased glycemic variability and the use of high-dose SUs prolonged the duration of severe hypoglycemia. In patients treated with SUs, treatments should be adjusted considering possible large glycemic variability and the development of hypoglycemia, regardless of HbA1c levels or TIR values.

## Supplementary Information

Supplementary Information

## Data Availability

All data generated or analysed during this study are included in this published article and its Supplementary information file.
