# Supplementary material for: Enlarged glycemic variability in sulfonylurea-treated well-controlled type 2 diabetics identified using continuous glucose monitoring
Source: Sci Rep. 2021 Mar 1;11:4875. doi: 10.1038/s41598-021-83999-z (PMC7921550; doi:10.1038/s41598-021-83999-z)
Supplement: Supplementary file 1 — Supplementary Information [file 41598_2021_83999_MOESM1_ESM.docx]

**Enlarged glycemic variability in sulfonylurea-treated well-controlled type 2 diabetics identified using continuous glucose monitoring**

Fumi Uemura^1^, Yosuke Okada^1^, Keiichi Torimoto^1^, and Yoshiya Tanaka^1^*

^1^First Department of Internal Medicine, School of Medicine, University of Occupational and Environmental Health, Japan, Kitakyushu, Japan

**Supplementary information**

Supplemental Table S1

Supplemental Table S2

**Supplemental Table S1.** Linear multivariable analysis with Time below range <54 mg/dL as the dependent variable.

|  | Univariable linear regression | | | Multivariable linear regression | | |
| --- | --- | --- | --- | --- | --- | --- |
|  | β | SE | P | β | SE | P |
| Age | 0.004 | 0.004 | 0.292 | 0.001 | 0.005 | 0.830 |
| Gender | -0.06 | 0.111 | 0.592 | -0.056 | 0.122 | 0.647 |
| BMI | -0.013 | 0.009 | 0.188 | -0.013 | 0.011 | 0.246 |
| HbA1c(NGSP) | -0.008 | 0.036 | 0.816 | -0.012 | 0.041 | 0.773 |
| eGFR | 0.00033 | 0.002 | 0.887 | 0.002 | 0.003 | 0.597 |
| Micro-angiopathy | 0.038 | 0.113 | 0.736 | 0.077 | 0.121 | 0.526 |
| Macro-angiopathy | 0.117 | 0.149 | 0.434 | 0.145 | 0.164 | 0.379 |
| SU | 0.215 | 0.109 | 0.050 | 0.179 | 0.12 | 0.139 |
| TZD | 0.226 | 0.145 | 0.122 | 0.133 | 0.156 | 0.397 |
| BG | -0.183 | 0.112 | 0.104 | -0.23 | 0.125 | 0.069 |
| α-GI | -0.123 | 0.179 | 0.494 | -0.258 | 0.207 | 0.214 |
| Glinide | -0.112 | 0.437 | 0.798 | -0.167 | 0.499 | 0.738 |
| DPP4 inhibitor | -0.14 | 0.113 | 0.217 | -0.208 | 0.128 | 0.108 |
| GLP1RA | -0.114 | 0.311 | 0.715 | -0.221 | 0.328 | 0.501 |
| SGLT2 inhibitor | -0.111 | 0.615 | 0.857 | 0.003 | 0.646 | 0.996 |
| R^2^ |  | | | 0.123 | | |

We used univariable and multivariable linear regression analyses to estimate regression coefficients for changes in Time below range <54 mg/dL. Age, gender, BMI, HbA1c(NGSP), eGFR, microangiopathy, macroangiopathy, SU, TZD, BG, α-GI, glinide, DPP4 inhibitors, GLP1RA, and SGLT2 inhibitors were fed into the model. We selected one of similar indicators from which multicollinearity may occur for each factor. Neuropathy, retinopathy, and nephropathy were evaluated as micro-angiopathy. β, regression coefficient; SE, standard error.

**Supplemental Table S2.** Linear multivariable analysis with Time below range <54 mg/dL as the dependent variable.

|  | Univariable linear regression | | | Multivariable linear regression | | |
| --- | --- | --- | --- | --- | --- | --- |
|  | β | SE | P | β | SE | P |
| Age | 0.004 | 0.004 | 0.292 | 0.005 | 0.005 | 0.601 |
| Gender | -0.06 | 0.111 | 0.592 | -0.061 | 0.119 | 0.610 |
| BMI | -0.013 | 0.009 | 0.188 | -0.012 | 0.011 | 0.274 |
| HbA1c(NGSP) | -0.008 | 0.036 | 0.816 | -0.07 | 0.040 | 0.852 |
| eGFR | 0.00033 | 0.002 | 0.887 | 0.002 | 0.003 | 0.552 |
| Micro-angiopathy | 0.038 | 0.113 | 0.736 | 0.062 | 0.120 | 0.609 |
| Macro-angiopathy | 0.117 | 0.149 | 0.434 | 0.103 | 0.161 | 0.524 |
| High-dose SU | 0.560 | 0.196 | 0.005* | 0.487 | 0.219 | 0.028* |
| TZD | 0.226 | 0.145 | 0.122 | 0.078 | 0.157 | 0.620 |
| BG | -0.183 | 0.112 | 0.104 | -0.212 | 0.124 | 0.089 |
| α-GI | -0.123 | 0.179 | 0.494 | -0.342 | 0.208 | 0.103 |
| Glinide | -0.112 | 0.437 | 0.798 | -0.109 | 0.492 | 0.826 |
| DPP4 inhibitor | -0.14 | 0.113 | 0.217 | -0.174 | 0.128 | 0.178 |
| GLP1RA | -0.114 | 0.311 | 0.715 | -0.134 | 0.325 | 0.681 |
| SGLT2 inhibitor | -0.111 | 0.615 | 0.857 | -0.067 | 0.631 | 0.916 |
| R^2^ |  | | | 0.123 | | |

We used univariable and multivariable linear regression analyses to estimate regression coefficients for changes in Time below range <54 mg/dL. Age, gender, BMI, HbA1c(NGSP), eGFR, microangiopathy, macroangiopathy, High-dose SU, TZD, BG, α-GI, glinide, DPP4 inhibitors, GLP1RA, and SGLT2 inhibitors were fed into the model. We selected one of similar indicators from which multicollinearity may occur for each factor. Neuropathy, retinopathy, and nephropathy were evaluated as micro-angiopathy. β, regression coefficient; SE, standard error. * p < 0.05
